# Energy Transfer from Magnetic Iron Oxide Nanoparticles: Implications for Magnetic Hyperthermia

**DOI:** 10.1021/acsanm.3c01643

**Published:** 2023-05-17

**Authors:** Gloria Tabacchi, Ilaria Armenia, Giovanni Bernardini, Norberto Masciocchi, Antonietta Guagliardi, Ettore Fois

**Affiliations:** † Dipartimento di Scienza e Alta Tecnologia (DSAT), University of Insubria, and INSTM, Via Valleggio 11, I-22100 Como, Italy; ‡ Instituto de Nanociencia y Materiales de Aragón (INMA), CSIC-Universidad de Zaragoza, Zaragoza 50009, Spain; § Dipartimento di Biotecnologie e Scienze della Vita (DBSV), University of Insubria, Via Dunant 3, I-21100 Varese, Italy; ∥ Istituto di Cristallografia − To.Sca.Lab and INSTM, CNR, Via Valleggio 11, I-22100 Como, Italy

**Keywords:** magnetic iron oxide, magnetic nanoparticle hyperthermia, density functional calculations, X-ray diffraction, nanoparticles

## Abstract

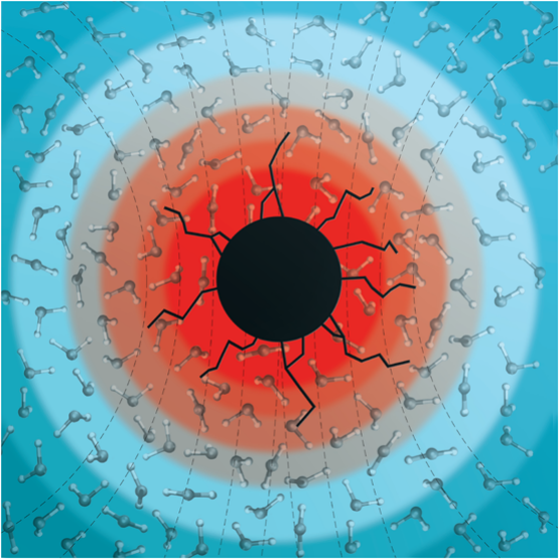

Magnetic iron oxide nanoparticles (IONPs) have gained momentum in the field of biomedical applications. They can be remotely heated via alternating magnetic fields, and such heat can be transferred from the IONPs to the local environment. However, the microscopic mechanism of heat transfer is still debated. By X-ray total scattering experiments and first-principles simulations, we show how such heat transfer can occur. After establishing structural and microstructural properties of the maghemite phase of the IONPs, we built a maghemite model functionalized with aminoalkoxysilane, a molecule used to anchor (bio)molecules to oxide surfaces. By a linear response theory approach, we reveal that a resonance mechanism is responsible for the heat transfer from the IONPs to the surroundings. Heat transfer occurs not only via covalent linkages with the IONP but also through the solvent hydrogen-bond network. This result may pave the way to exploit the directional control of the heat flow from the IONPs to the anchored molecules—i.e., antibiotics, therapeutics, and enzymes—for their activation or release in a broader range of medical and industrial applications.

## Introduction

Owing to their unique properties, magnetic nanoparticles (NPs) are intensively studied for potential uses in biomedical technologies^1^ such as medical imaging, magnetofection, magnetically triggered drug release, and cancer therapy.^2−4^ In these applications, biomolecules,^5^ like nucleic acids,^6,7^ antibodies,^8^ sugars,^9−11^ antibiotics,^12,13^ and enzymes,^14−17^ are connected to magnetic NPs by either surface adsorption or covalent binding. Whereas static magnetic fields drive the NP-linked biomolecules to selected areas, alternating magnetic fields (AMF) transfer energy to the NPs by Néel or Brownian relaxation.^18^ The thermally activated NPs, in turn, transfer heat to the surrounding. Such an ability to generate local heating—known as magnetic NP hyperthermia (MNH)—holds promise in nanomedicine as an innovative cancer treatment.^1,3^ Beyond biomedical applications, MNH is currently exploited in chemical and materials science as well.^19^

To optimize MNH in nanoscience applications, it would be crucial to understand how AMF-generated heat is transferred from the NP toward the environment. However, to the best of our knowledge, the energy-transfer mechanism from the NP to the surrounding molecules is still debated.^20^ Indeed, previous studies suggested that, at the nanoscale regime, the reliability of the generally accepted Fourier heat transfer mechanism—valid at the macroscopic scale—becomes questionable.^21^ Actually, a crucial MNH feature is its local nature, as its effects are significant only at the nanometer scale. More specifically, at low NP concentration, the heating is localized in a few nanometers range from the NPs and does not affect the overall temperature of the medium.^22^ Such a local nature of MNH is supported by experiments indicating that, in magnetically heated iron oxide NPs, the temperature measured by moving apart from the NP surface decays faster than as predicted by the diffusive Fourier law.^23^

In this scenario, some of us recently made use of the local character of MNH by showing that two enzymes with different temperature optima (∼80 and ∼25 °C, respectively) worked properly in the same reaction pot.^24^ The high T enzyme was covalently linked to magnetic NPs and activated in a wireless fashion via AMF,^24,25^ indicating a local temperature increase close to the NP surfaces.^23,26^ Notably, even the other enzyme, with much lower temperature optima but not linked to the NPs, could optimally function in the same reaction pot for several minutes. These results indicate the onset of an AMF-induced negative temperature gradient around the activated NPs.^24,26^

Understanding the phenomena governing the transfer of the heat generated by the NPs would allow their better engineering, especially for biomedical applications. Indeed, it would be possible to harness the energy flux in a smarter way to activate the immobilized enzyme or to induce the release of the molecule of interest. In this context, iron oxide-based NPs (IONPs) are preferable because their biocompatibility is superior to that of other magnetic NPs, such as cobalt- and nickel-oxide-based NPs.^27^ Thus, we will focus on iron oxide NPs functionalized with a commonly used anchoring group. Indeed, a winning strategy to covalently bind, e.g., enzymes to IONPs, is to use, as anchoring groups, species like amino-propyl-triethoxysilane (APTES), which can form bridges between hydroxylated oxide surfaces and (bio)molecules and even with living systems.^28^ We address the heat transfer issue from the NPs to the surroundings by first investigating the structure, stoichiometry, and defectiveness of the magnetic NPs via synchrotron radiation and wide-angle X-ray total scattering (WAXTS) analysis^29^ and then by analyzing the behavior of APTES-functionalized IONPs in water by first-principles modeling.

Our aim here is to shed light, at the atomistic level, on the mechanism of the unconventional heat transfer process from an AMF-heated magnetic NP to the surrounding molecules. Recently, a first-principles molecular dynamics approach, coupled with linear response theory, allowed the efficient calculation of heat transport coefficients for bulk (macroscopic) systems.^30−32^ However, such an approach rests on the diffusive mechanism, which is typical of the classical Fourier law for heat transport; hence, it is not suitable for a nanometer-scale problem. Theoretical investigations on non-Fourier heat transport at the nanoscale have been recently reviewed.^33,34^ Nevertheless, most of these studies were mainly focused on simple low-dimensionality systems—such as, e.g., atomic or molecular one-dimensional chains, or graphene-like two-dimensional structures—while, conversely, both the system and the process considered in the present study are quite complex. Indeed, the system is a magnetic NP covalently linked to the APTES anchoring group and solvated by water, i.e., a nanosized three-dimensional magnet exhibiting a solid–liquid interface. In addition, as shown by the abovementioned experiments, the magnetically induced heat transfer process from the NPs to the surrounding species is “local”, being effective only at the nanometer scale. These issues—which are difficult to be addressed via a macroscopic approach—may be conveniently tackled by atomistic scale modeling. Since the system to be modeled is magnetic, and it is not known at the outset whether the magnetization affects also the covalently anchored species, the use of a methodology that can accurately calculate the magnetization is mandatory. In particular, first-principles simulations—which are based on a quantum-mechanical description of the electronic structure of the system—are the method of choice in our case. The price to pay is that this computationally demanding approach limits the size of the affordable models, preventing therefore a direct estimation by first-principles of the decay profile of MNH effects. Nevertheless, the chosen strategy may provide fundamental insight into the mechanism by which magnetically induced heat may be transferred from the atoms of the IONPs to those of the anchoring group and/or to the water molecules in the immediate proximity of the magnetic NP.

Thus, as strongly suggested by both experimental evidence and theoretical considerations, methodologies relying on diffusive-based theories for heat transport could not be applicable in the present case. Therefore, rather than calculating first-principles Fourier heat transport coefficients (which are pertinent to the macroscopic regime) or adopting other diffusion-based approaches (e.g., the Boltzmann transport equation), in this work we explore whether different microscopic mechanisms, not directly connected to the classical (diffusional) heat transport theories, may be operative in MNH effects. For all of these reasons, to investigate the thermal behavior of a magnetic NP, covalently linked to APTES, we have adopted an approach based on equilibrium first-principles molecular dynamics and on the spectral analysis of atomic vibrational motions via the linear response theory.

We anticipate here that our results reveal that, at low wavenumbers in the infrared region, the vibrational spectra of the “magnetic” Fe cations of the NPs overlap with the vibrational spectra of the atoms surrounding the magnetic NPs. This finding suggests that the heat flow from the NPs to the atoms in the proximity of the NP surface may be governed by a resonance mechanism,^35^ which, typically, operates at the nanometer scale.

## Experimental Section

### Synthesis and Functionalization of IONPs

IONPs were synthesized by coprecipitation method, as previously reported by Balzaretti et al.^15^ A 380 mL solution was prepared with 87 mM FeCl_3_ and 42 mM FeCl_2_, left stirring for 30 min by adding 1.5 mL of 37% HCl to completely dissolve the salts. The addition of 25 mL of a solution of NH_4_OH 25% allowed the nucleation centers to form the IONPs. Particles were washed several times with MilliQ water, and 40 mL of 2 M HNO_3_ was added and heated at 90 °C for 5 min. The particles were separated by a magnet from the reaction mixture; then, 60 mL of 0.34 M solution of Fe(NO_3_)_3_·9 H_2_O was added. The IONPs were heated to 90 °C for 30 min and washed three times. Finally, the IONPs were suspended in 50 mL of MilliQ water and dialyzed overnight. IONPs were stored at 4 °C [see the Supporting Information for further details].

To introduce amino groups on the surface and stabilize the IONPs, a 1 mL of 1.5 M solution of APTES in ethanol was added to 150 mg of IONPs and stirred for 1 h at room temperature. Then, the temperature was increased to 90 °C and stirred for an additional hour. The APTES-coated IONPs were collected by centrifugation and washed several times and suspended in MilliQ water.

### Synchrotron X-ray Data Collection and Wide-Angle Total Scattering Analysis

Synchrotron wide-angle X-ray total scattering (WAXTS) data were collected on stable colloidal aqueous suspensions of both APTES-functionalized and unfunctionalized IONPs at the MS-X04SA beamline of the Swiss Light Source of the Paul Scherrer Institute (Villigen, CH).^36^ Suspensions were loaded into glass (Hilgenberg GmbH G50) capillaries, 0.8 mm in diameter, and measured, while spinning at ca. 2 Hz, using a Debye–Scherrer transmission geometry, 22 keV X-ray photons, and the single-photon MYTHEN-II detector covering 120° with 0.0036° resolutions.^37^ The operational wavelength (λ = 0.563626 Å) was calibrated against the standard Silicon SRM 640c powder certified by NIST. Transmission and scattering data were acquired on IONPs, IONPs-APTES, and their “blank” (IONPs-free) solutions. Using a beamline-defined protocol inspired by the work of Ritter et al.,^38,39^ angle-dependent intensity corrections were applied to the raw data to account for sample absorption effects. Air and (absorption-corrected) capillary scattering contributions, independently measured, were also subtracted. In addition, using the certified silicon powder as an external standard, zero angle and *x*, *y* capillary offsets to the 2θ values were applied.

Data analysis relied on the use of the Debye function analysis^40^ in its fast formulation^41^ as implemented in the Debussy Suite,^42^ enabling both Bragg and diffuse scattering from the sample to be treated on an equal basis, according to a total scattering approach in reciprocal space. We employed atomistic models of Fe*_x_*O_4_ nanosized clusters, described in *Fd*-3*m* with (refinable) iron vacant sites, allowing any composition from magnetite, Fe_3_O_4_, to maghemite, γ-Fe_2_O_3_, to be managed.^43^

A population of spherical nanocrystals of increasing diameter (up to a maximum value of 40 nm) was generated, and sample polydispersity was modeled according to a log–normal size distribution function, a model that is found to be valid for many colloidal samples.^44^ Model parameters were optimized against the experimental WAXTS data, performed by the Debussy code.^42^ The analysis enabled the accurate determination of the unit cell parameter, the sample stoichiometry, the average crystal size, and its variance. The numerical analysis provided the results presented in the Results and Discussion section and a few relevant plots, which are inserted in the Supporting Information, jointly with a more extended description of the Debye function analysis-based method of analysis for nanocrystalline materials and the overall modeling of the investigated nanoparticles.

### First-Principles Modeling

Structural information gathered from the WAXTS data was used to build the atomistic model of the system to be simulated. The iron oxide was modeled by adopting a slab geometry, the stoichiometry of which is Fe_2.60_O_4_, close to both the maghemite ideal stoichiometry Fe_2.667_O_4_ and to the experimentally detected (Fe_2.66_O_4_) one (see below). The oxide slab consisted of 36 Fe occupying octahedral sites and 16 Fe in tetrahedral sites. The chosen model slab exposes the (111) facet of maghemite and was partially hydroxylated at the surfaces. The iron oxide slab stoichiometry was [Fe_52_O_76_(OH)_4_]. Octahedral site vacancies were randomly distributed in the slab; however, in line with experimental indications (see the Results and Discussion section), vacancy close contacts were avoided. The maghemite slab was built by adopting the cell parameters determined from the synchrotron X-ray diffraction experiments. The slab area was 10.234 × 11.817 Å^2^ (in the *x*, *y* plane), and the thickness was 14 Å in the *z-*direction.

In order to mimic a chemisorbed APTES molecule, a-Si(OCH_3_)_2_-(CH_2_)_3_NH_3_^+^ residue was linked to the hydroxylated slab surface, forming a Fe–O–Si bridge. As in the experimental conditions, the terminal amino group is protonated (−NH_3_^+^). The ammonium group is indeed relevant as it could be exploited as an anchoring center for (bio)molecules.

**Figure 1:**
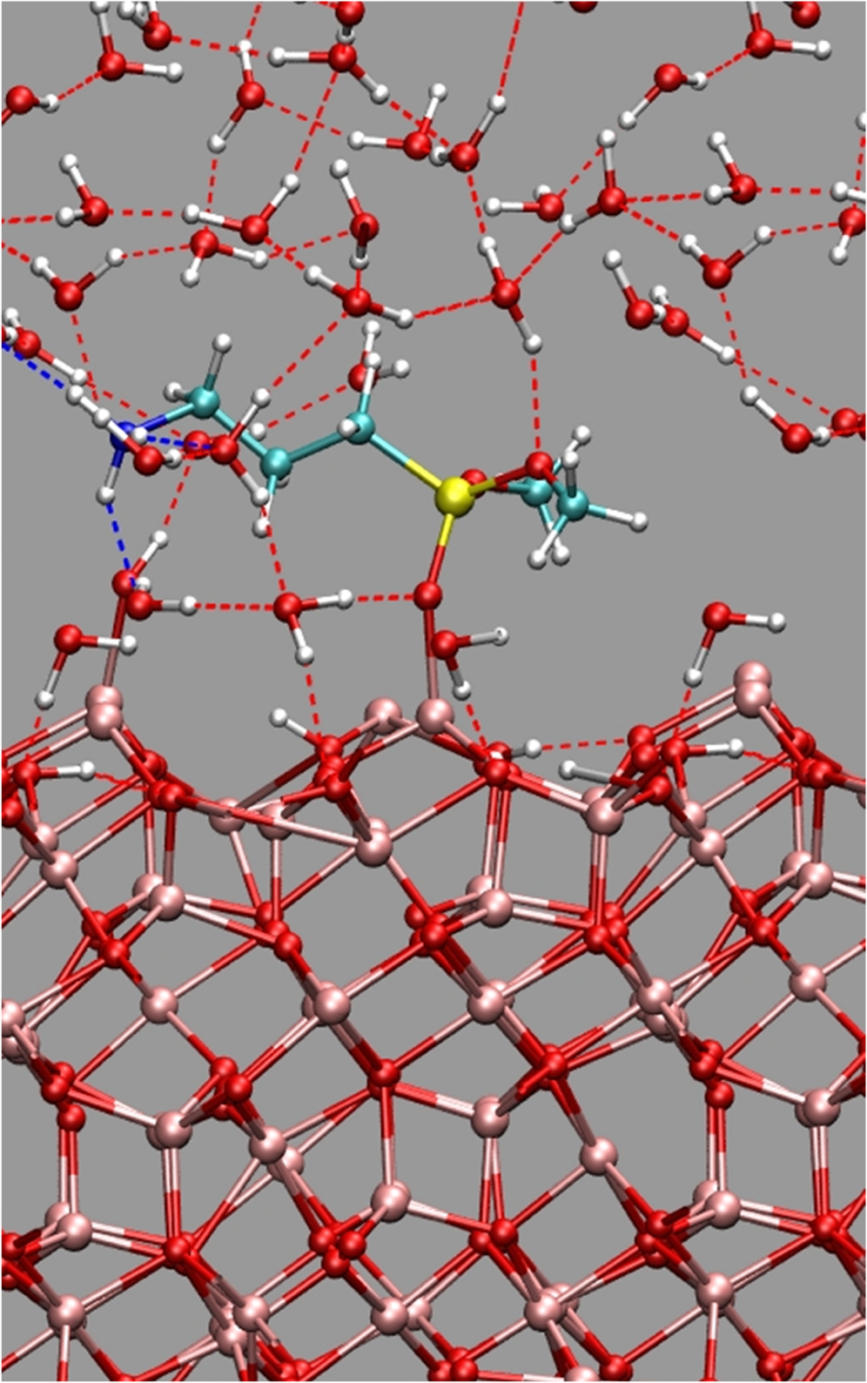
Hydrated IONP-APTES model. Atom colors; Fe, pink; O, red; H, white; Si, yellow; C, cyan; N, blue, H-bonds, dotted line.

A vacuum region of 12 Å was added along the *z*-direction of the slab and filled with water molecules. Hence, the orthorhombic simulation box was 10.234 × 11.817 × 26.0 Å^3^. Specifically, the structure of the chemisorbed −Si(OR)_2_–(CH_2_)_3_NH_3_^+^ moiety on the maghemite slab was solvated with 41 water molecules per simulation cell. The number of water molecules was chosen to reproduce as closely as possible the density of liquid water at ambient conditions. Periodic boundary conditions were applied in three dimensions to the simulation cell, which contained a total of 284 atoms (52 Fe, 124 O, 101 H, 1 N, 5 C, 1 Si). The stoichiometry of the periodically repeated simulation cell was [Fe_52_O_76_(OH)_4_]-Si(OCH_3_)_2_-(CH_2_)_3_-NH_3_·(H_2_O)_41_, The entire system is electrically neutral and is graphically represented in Figure 1.

The electronic structure of the model was studied by spin-unrestricted density functional theory with empirical dispersion,^45,46^ an approach that reproduced satisfactorily the physicochemical behavior at oxide surfaces.^47−52^ To gain insight into the NP magnetic properties, we computed the magnetization self-consistently by the Hubbard Hamiltonian approach^53^ appropriate for magnetic oxides.^54,55^ Then, we performed first-principles molecular dynamics at 298 K^56−58^ in the NVT ensemble. Along the molecular dynamics trajectory, the magnetization was kept fixed to the value obtained via the Hubbard Hamiltonian approach (see the Supporting Information for further details).

In principle, an AMF should be introduced as a perturbation in the Hamiltonian of the system. However, by invoking linear response theory, it is not necessary to consider such a perturbation explicitly since the “response” of the system may be simulated by adopting the unperturbed Hamiltonian and using appropriate time-correlation functions. Moreover, the use of linear response theory is supported by experimental evidence: in particular, it has been shown that, in IONPs, the temperature increases linearly with the intensity of the applied AMF.^23^ In the general framework of linear response theory, for a given time-dependent observable *A*(*t*) (*t* stands for time), its correlation function *C*(*t*) is defined as *C*(*t*) = ⟨*A*(*t*)*A*(0)⟩, where the ⟨ ⟩ symbol indicates time average.^59,60^ In this work, time-correlation functions were obtained by correlating nuclei velocities or nuclei momenta from the calculated 15 ps trajectory. Spectral analysis of atomic motion was obtained by calculating power spectra via the Fourier transforms (FTs) of such correlation functions.^59,60^ Two different kinds of correlation functions were considered, namely velocity–velocity autocorrelation function (VVACF) and momentum–momentum cross-correlation functions (MMCCFs). From FT of VVACF, it is possible to gain indications of the oscillating frequencies of a given atom. MMCCF is the correlation of the projection of the momentum of an atom on the momentum of a different atom. The MMCCF provides indications of momentum transfer from an atom to another one,^61,62^ while its FT indicates the frequencies at which such momentum transfer occurs.

## Results and Discussion

Pristine iron oxide NPs (labeled IONP) and APTES-functionalized NPs (labeled IONP-APTES) were prepared as described by Balzaretti et al.^15^ For this kind of materials, the amount of heat generated by the magnetic nanoparticles can be analyzed by their specific absorption rate (SAR) when applying an alternating magnetic field. SAR is defined as the amount of power absorbed by the sample per unit mass (W/g). In the case of noncoated and APTES-coated NPs, the SAR values were examined at 710 kHz and 300 Gauss, using the D500 series (Nanoscale Biomaterial) and particle concentration of 1 mg_Fe_/mL in MilliQ water. A loss of the heating capacity was observed after coating with APTES; indeed, the SAR value for IONPs is 393 ± 9 W/g, whereas for IONP-APTES, it is 242 ± 13 W/g.^24^

The WAXTS analysis, performed by the Debye scattering equation method using the Debussy code,^42^ enabled the accurate determination of the unit cell parameters, the sample stoichiometry, and the average NP size and variance for the two iron oxide samples, as listed in Table 1.

**Table 1:** Structural and Microstructural Parameters for the IONP and IONP-APTES Samples Obtained from WAXTS Analysis^a^

sample	cell (Å)	⟨*D*⟩_N_, σ_N_ (nm)	⟨*D*⟩_M_, σ_M_ (nm)	s.o.f.	*x*, s.o.f.	*x*, cell
IONP	8.3561	2.1, 1.9	11.0, 7.7	0.83	2.66	2.73
IONP-APTES	8.3561	1.9, 1.8	11.6, 8.2	0.84	2.66	2.73

a ⟨*D*⟩_N_ and ⟨*D*⟩_M_ are the NP mean diameter values from number- and mass-based distributions, respectively, with variances σ_N_ and σ_M_. *x* addresses the experimental stoichiometry in the standard Fe*_x_*O_4_ formulation, as obtained from s.o.f. or from cell axis calibration (see the Supporting Information).

The results reveal that the two samples are essentially Fe(III) oxide of the γ-Fe_2_O_3_ type, i.e., maghemite. Taking magnetite as the reference structure, upon full iron oxidation to maghemite, vacant sites take place inside the crystal (because of charge balance issues).

According to the way vacancies self-organize inside the structure, maghemite can exhibit fully disordered vacancies (crystallizing in the cubic F lattice, space group *Fd*-3*m*),^63^ a partially ordered system (cubic P, space group *P*4_3_32, and its enantiomorph),^64^ or the vacancy-ordered tetragonal supercell (space group *P*4_2_2_1_2^65,66^), though the latter was never observed in nanosized systems. In our case, the tiny “cubic P” superstructure peaks (110, 200, and 210), which are commonly measurable if intense and highly collimated synchrotron X-ray radiation is used, went unobserved, suggesting the occurrence of the (average) cubic F crystal phase with the highest vacancy disorder, where cross-talking between vacant sites is highly limited.

The correct (average) stoichiometry can be derived from the refined site occupancy factor (s.o.f.) of the vacant Fe octahedral site, using the information carried by the peak intensities, or, more reliably, from the accurate calibration curve proposed in ref (67), built using ca. 30 differently prepared IONP samples, characterized by the same WAXTS technique. The values collected in Table 1 witness not only the very small size of the prepared NPs but also a large spread of sizes, making each sample far from being monodisperse (here σ_M_/⟨*D*⟩_M_ ca. 60%, while polydispersity values well below 15% or so have been reported in highly controlled samples^68^).

The above structural data were used to build the slab model for the IONP-APTES system (see also the Supporting Information) where the organic moiety is covalently bonded to the IONP via an Fe–O–Si bridge. Only one of three plausible Si–O bonds between trialkoxysilane and the surface was considered because multiple bonds would lead to tensioned O–Si–O angles and high stresses to the APTES-surface linkage.^69^ The calculated magnetization was localized essentially on the Fe cations, and, in line with reported experiments on maghemite (γ-Fe_2_O_3_),^65^ tetrahedral and octahedral Fe centers exhibited opposite spins, while no spin polarization was detected on the anchored organic moiety. Remarkably, this result rules out a mechanism of direct transfer of the magnetization from the NP to the organic moiety.

During the simulation, the aminodialkoxysilane moiety remained stably bonded to the iron oxide surface as shown in Figure 1. In particular, the Fe–O and O–Si bond lengths averaged 1.91 ± 0.04 and 1.62 ± 0.03 Å, respectively, while the average Fe–O–Si bond angle was 138 ± 12°.

A closer analysis of the trajectory revealed a complex pattern of hydrogen bonds that connects the hydrophilic parts of the chemisorbed molecule with the NP surface via the solvating water molecules (see Figures 1, S2, and S3 and Movie S1). Such a hydrogen-bond pattern governs the dynamical behavior of the system, so it likely plays a role in the heat transfer mechanism as well. To confirm this hypothesis, we used linear response theory^59^ to calculate, for the atoms in the model system, time-correlation functions^61^ and their power spectra. With this procedure, correlations between the motions of specific atoms may be directly deduced from the comparison of the power spectra of these atoms, obtained by the Fourier transform of the pertaining time-correlation functions. For example, a significant overlap between the power spectra of two atoms indicates that the motions of these two atoms are highly correlated; hence, thermal energy could be transferred efficiently between these two atoms. Such a strategy, which has been already applied to study heat transfer in simple liquids,^62^ could therefore provide a microscopic view of the thermal behavior of the IONP, the surrounding water molecules, and the surface-linked APTES.

Overall, the power spectra of the velocity–velocity autocorrelation functions (VVACFs) show that the motions of the atoms in the model are highly correlated. Specifically, all power spectra present relatively high-intensity peaks in the 100–350 cm^–1^ region, which is close to kT (kT = 207 cm^–1^ at 298 K). This result shows that vibrational states characterized by such wavenumbers, typical of low energy modes, are well populated. Hence, in the solvated aminodialkoxysilane–IONP model system, atoms showing overlapping power spectra can efficiently transfer energy among each other by resonance.^35^

**Figure 2:**
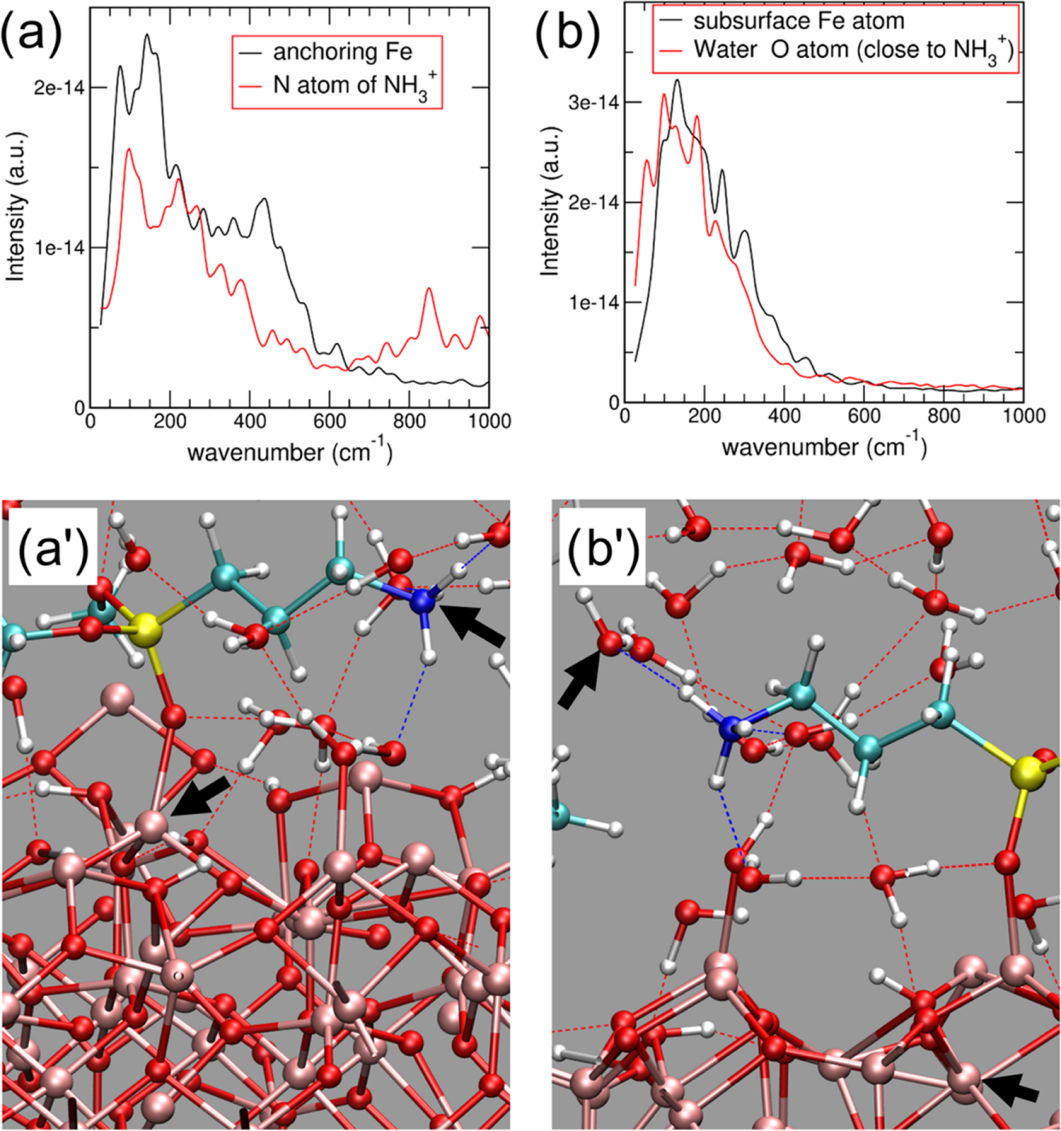
(a) VVACF power spectra for the anchoring Fe atom (black line) and the amino-group N atom (red line), indicated with a black arrow in panel (a′); (b) VVACF power spectra for a subsurface Fe atom (black line) and a water oxygen atom (red line) indicated with a black arrow in panel (b′). Atom color codes as in Figure 1.

Accordingly, the VVACF power spectra for the N atom of the amino group and for the IONP Fe cation linked to the alkoxysilane—which are relatively distant from each other but are connected by a covalent bond chain—significantly overlap in the 100–350 cm^–1^ region (Figure 2a,a′). Such wavenumbers correspond to a temperature range between 144 and 503 K, with significant overlap at 353 K (245 cm^–1^). This value is well in line with the optimal working temperatures monitored experimentally for the real enzyme–NP system under AMF (80 °C, i.e., 353 K).^24^ Interestingly, a similar behavior is also detected for the VVACF power spectra even for relatively distant atoms that are not connected through covalent bond chains—for example, a subsurface Fe atom of the NP and a water oxygen (see Figure 2b,b′).

**Figure 3:**
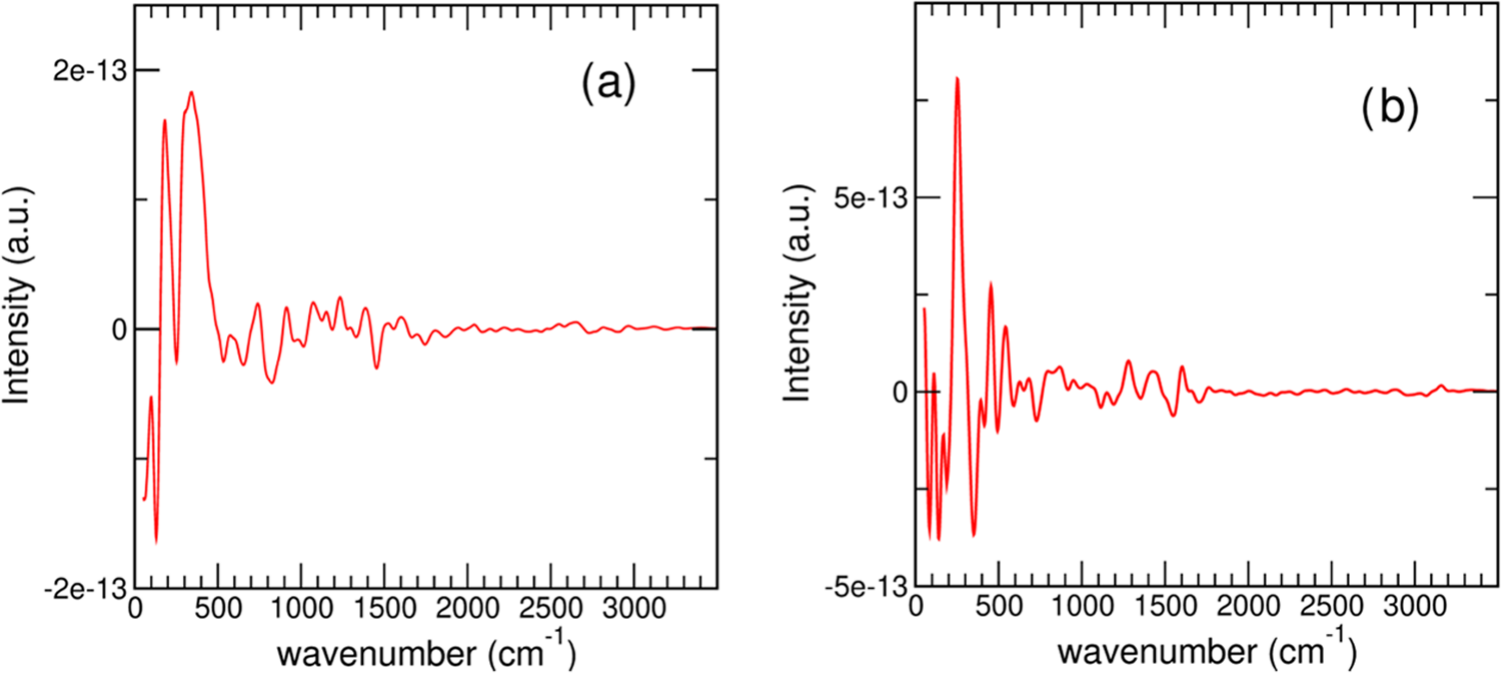
(a) Power spectrum of the MMCCF of the Fe atom of the Fe–O–Si bridge with the N atom of the amino group; (b) power spectrum of the MMCCF between the Fe atom involved in the Fe–O–Si bridge and a water molecule O atom lying at an average distance of 6.6 Å.

This result suggests that the energy flow from the IONP to the solvent, via the hydrogen-bond network, may be viable. To extend our analysis, we calculated power spectra from momentum–momentum cross-correlation functions (MMCCFs), which provide a direct probe for interatomic energy transfer.^61,62^Figure 3a shows the power spectrum for the momentum of the Fe atom linked to the O–Si bond cross-correlated with the momentum of the N atom. The strong peaks below 500 cm^–1^ indicate that energy can flow among the magnetically active atoms and the covalent backbone of the chemisorbed species. The MMCCF power spectrum for the same Fe atom cross-correlated with a water molecule oxygen (Figure 3b) provides a similar picture of energy flow between the IONP and the environment, this time through the hydrogen-bond network. Experiments indicate that in AMF-exposed IONPs, heating fades away within 3–4 nm from the NP surface.^23^ Such a range is typical of energy transfer by resonance whose efficiency decays rapidly with distance.^35^ Although the limited size of our model—imposed by the stringent necessity of using a quantum-mechanical description of the electronic structure—does not allow us to estimate a characteristic length within which the energy flow remains appreciable, this result shows that energy transfer among magnetically active atoms and solvent molecules at an average distance of 6.6 ± 0.2 Å could be significant (Figure 3b). Hence, our data support an energy flow mechanism through the hydrogen-bond network, definitely involving the first layers of solvent molecules. Therefore, two possible channels of energy flow exist: one directly connecting the magnetic atoms with the covalent backbone of the chemisorbed moiety, and the other one involving the hydrogen-bond network of the solvent and the NP surface. Both channels may be active in the AMF-induced local heating observed in the immediate (few nanometer) surroundings of magnetic NPs. This conclusion opens the intriguing question of whether the two channels might equally contribute to magnetically induced heating or rather one is predominant. Convincing experimental evidence settling this conundrum is still awaited.

## Conclusions

In summary, we propose a mechanism able to explain the capability of magnetic NPs in generating local heating in their surroundings, once remotely heated via AMF. In the present case, WAXTS analyses identified the nature of the magnetic IONPs, which consist of ferrimagnetic maghemite with disordered distribution of vacant sites. This allowed us to build an atomistic model of the IONP–alkoxysilane interface and to study its energy-transfer dynamics by first principles. We found that IONP atoms, organic bridge atoms, and water atoms oscillate at common frequencies in the 100–350 cm^–1^ region, thus enabling an efficient thermal energy transfer by resonance, leading to a newly proposed atomistic mechanism for AMF-induced local heating. When AMF is applied, IONPs heat up, thus locally increasing the population of IONP atom vibrational states; conversely, the vibrational states of both solvent and chemisorbed species are not directly affected by AMF. Then, a net energy flow via resonance occurs from the more populated states of the IONPs to the less populated states of the surroundings. Our findings underline that this energy flow may occur through both the atoms of the anchoring species and the solvent molecules. This insight may open the way to further investigations aimed at better understanding the roles and the relative importance of these microscopic energy flow pathways in MNH effects.

Finally, the microscopic mechanism governing energy flow from magnetic NPs here proposed for the first time may also foster its adoption in other yet unchartered fields involving the chemistry of molecules interacting with magnetic NP interfaces. Indeed, the study of this phenomenon with different coating materials opens the door to biocatalytic and biomedical applications where the coating molecule of the magnetic particle plays a key role.

## Supporting Information Available

Supporting Information on sample preparation and experimental details (ST1); Supporting Information for Debye function analysis with Figure S1 (plots of the Debye function analysis) (ST2); Supporting Information on first-principles modeling and computational details, including Figures S2 and S3, describing structural data from the first-principles molecular dynamics simulation (ST3 and ST4); and references relative to the supporting information (PDF)
Movie representing the time-evolution of the simulated system and atomic color codes (MP4)
