# Supplementary material for: Energy Transfer from Magnetic Iron Oxide Nanoparticles: Implications for Magnetic Hyperthermia
Source: ACS Appl Nano Mater. 2023 May 17;6(14):12914–21. doi: 10.1021/acsanm.3c01643 (PMC10391739; doi:10.1021/acsanm.3c01643)
Supplement: Supplementary file 1 — an3c01643_si_001.pdf [file an3c01643_si_001.pdf]

SUPPORTING INFORMATION for:

Energy Transfer from Magnetic Iron Oxide Nanoparticles: Implications for Magnetic Hyperthermia

Gloria Tabacchi,[a] Ilaria Armenia,[b] Giovanni Bernardini,[c] Norberto Masciocchi,[a] Antonietta Guagliardi,[d] Ettore Fois[a*]

[a.] *DSAT, University of Insubria, and INSTM, via Valleggio 11,-22100 Como, Italy. E-mail: ettore.fois@uninsubria.it*
[b] *Instituto de Nanociencia y Materiales de Aragón (INMA), CSIC-Universidad de Zaragoza, 50009 Zaragoza, Spain*
[c] *DBSV, University of Insubria, via Dunant 3, 21100 Varese, Italy.*
[d.] *Istituto di Cristallografia - To.Sca.Lab. and INSTM, CNR. via Valleggio 11, 22100 Como, Italy.*

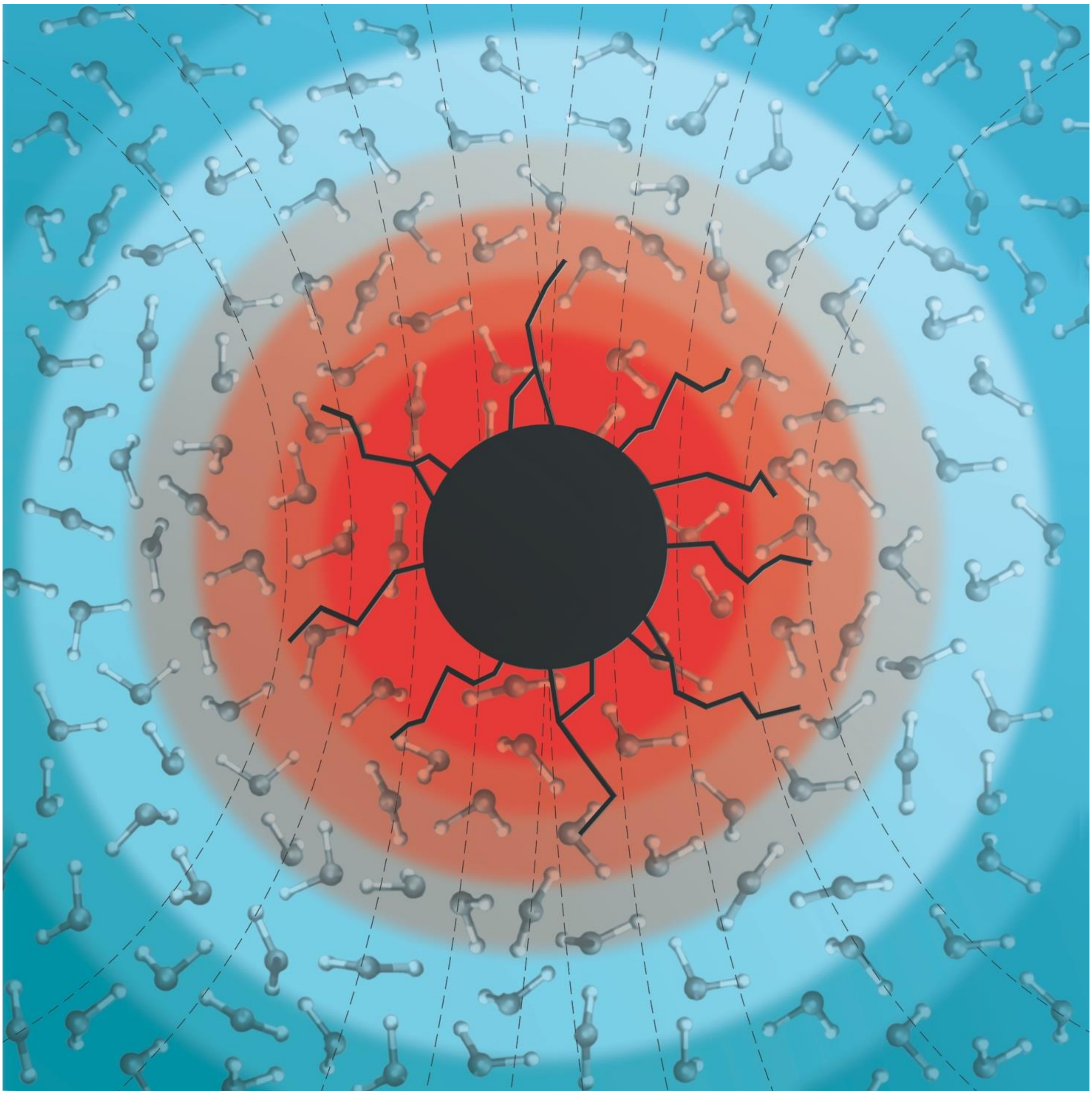

1. Supporting Text ST1-ST4
2. Supporting Figures S1-S3
3. Supporting Movie
4. Supporting References

Supporting Text ST1

Sample preparation and experimental details

Iron oxide NPs (IONP) were prepared according to the co-precipitation method reported by Balzaretti et al.[1] Briefly, 8.89 g of $FeCl_3 \times 6\ H_2O$ and 3.28 g $FeCl_2 \times 4\ H_2O$ were mixed in 380 mL of water, while dropping 1.5 mL of a solution of 37% HCl. The solution was maintained under a vigorous stirring for 30 min. Then, 25 mL of 25% NH_4OH were added and stirred vigorously for 10 min. The obtained NPs were washed three times with MilliQ water and then suspended in 40 mL of 2 M HNO_3. The suspension temperature was increased and heated at 90 °C for 5 min. Particles were magnetically separated and 60 mL of 0.34 M solution of $Fe(NO_3)_3 \times 9\ H_2O$ were added in order to obtain stable iron oxide γ-phase (maghemite) from magnetite NPs by reducing the Fe^{2+}/Fe^{3+} ratio.[2] The suspension was heated at 90 °C for 30 min under magnetic stirring. The NPs were collected by a magnet and suspended in MilliQ water and left in dialysis overnight.
The separations were performed using a strong magnet (NdFeB-magnet), while the magnetic stirring was performed using a weaker magnet. The few NPs that remained attached on the surface of the magnetic bar during the stirring could be easily recovered by washing with MilliQ water.
For APTES functionalization, a 1.5M solution of APTES, suspended in ethanol, was poured into 150 mg of IONPs suspended in MilliQ water. The reaction was maintained under mechanical stirring for 1 h at room temperature and for 1 h at 90 °C. The amino-modified NPs (IONP-APTES) were washed three times with MilliQ water, separated by centrifugation and suspended in MilliQ water. The obtained NPs were stored at 4 °C.

Supporting Text ST2

The Debye Function Analysis

In order to investigate the variability of the lattice parameters and of the cation vacancies in magnetite–maghemite nanoparticles (NPs) with their size-dependent oxidation we used a modelling approach relying on the Debye scattering equation[3] in the formulation recently proposed by Cervellino et al.[4]:

$$I(Q) = \sum_{j=1}^{N} f_j(Q)^2 o_j^2 + 2 \sum_{j>i=1}^{N} f_j(Q) f_i(Q) T_j(Q) T_i(Q) o_j o_i \sin(Q d_{ij})/(Q d_{ij})$$

where $Q = 2\pi q$, $q = 2\sin\theta/\lambda$ is the length of the reciprocal scattering vector, λ is the radiation wavelength, f_j is the atomic form factor, T_j and o_j are (adjustable) atomic Debye–Waller and site occupancy factors (s.o.f's), d_{ij} is the interatomic distance between atoms *i* and *j*, and N is the number of atoms in the NP. Atomistic models of pure Fe_3O_4 nanocrystals of spherical shape were generated following a concentric shells model to build up a discrete population of NPs of increasing radius, *r*, of subsequent spheres with Δr equal to 0.328 nm [details on atomistic model construction of spherical nanocrystals can be found in Cervellino et al.[4]] Specific details on IONPs modelling and analysis can be found in Ref.[5] The long-known problem of extremely heavy computing time required by the Debye equation was solved by sampling the interatomic distances according to the algorithm described by Cervellino et al.[6] After calculating and storing the set of sampled distances of each nanocrystal for the entire nanoparticle population, the Debye pattern model of each sample was calculated and further adjusted to the experimental one by optimizing the following parameters: (i) the average ($<D>_N$) and standard deviation σ_N of a lognormal function, adopted to describe the nanocrystals size polydispersity. The adoption of this function is justified by experimental observations[7] and several theoretical models developed for single-process-driven NP syntheses;[8–10] (ii) the site occupancy factor (s.o.f.) of Fe ion in the octahedral site and (iii) the cubic unit cell parameter, both modeled according to a size-independent law,[5] and (iv) the isotropic Debye–Waller factor of each atom. Parameter optimization was performed through the simplex method.[11] The solvent signal was added as an additionally model "blank" component and scaled to the experimental data by least squares. For both the investigated samples, the best fits are shown in Fig. S1 which include also the entire log-normal size-distributions derived therefrom.

Supporting Figure S1

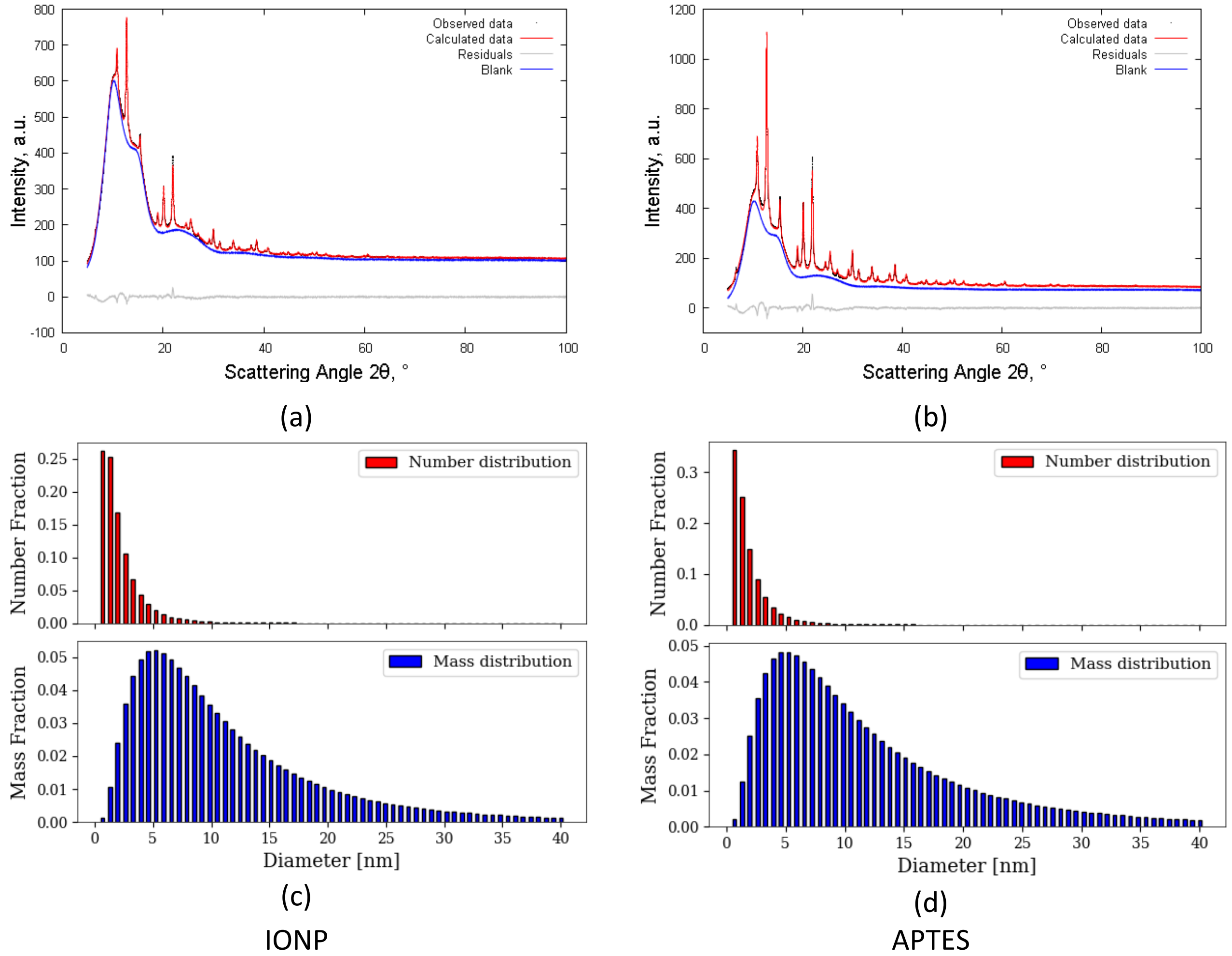

Figure S1. Final Plots of the Debye Functional Analysis performed on IONP (a) and IONP-APTES (b) samples. Final Goodness of Fit values were 2.67 and 3.88, respectively. In (c) and (d) the Number (red) and Mass (blue) size distributions derived therefrom, according to a lognormal function, are plotted.

Supporting Text ST3

First-principles Modeling

The magnetic IONP was modeled adopting a slab geometry, the stoichiometry of which is $Fe_{2.60}O_4$, close to both the maghemite ideal stoichiometry $Fe_{2.667}O_4$ and to the experimentally detected ($Fe_{2.66}O_4$) one (see main text). The oxide slab consisted of 36 Fe occupying octahedral sites and 16 Fe in tetrahedral sites. Octahedral site vacancies were randomly distributed avoiding close contacts among vacancies, which are energetically unfavorable. Structural information gathered from the WAXTS data were adopted to build the model slab. The chosen model slab exposes the (111) facet of maghemite and, in order to mimic a chemisorbed APTES model, a $-Si(OCH_3)_2-(CH_2)_3-NH_3^+$ residue was covalently bonded to the hydroxylated slab surface, forming a Fe-O-Si bridge. Forty-one water molecules completed the model (see Figure 1 of the main text) with a total stoichiometry of $[Fe_{52}O_{76}(OH)_4](Si(OCH_3)_2-CH_2-CH_2-CH_2-NH_3)41(H_2O)$. As in the experimental conditions, the terminal amino group is protonated ($-NH_3^+$). This charged group is indeed relevant as it could be exploited as anchoring centre for biomolecules.

Computational details

The simulation system consisted of a water-solvated $Si(OR)_3-(CH_2)_3NH_3^+$ ($R=CH_3$) moiety at the (111) facet of the maghemite slab. By considering chemisorption, one alkoxide –(OCH_3) group was removed and the Si was manually linked to a surface oxygen. The maghemite slab was characterized by a stoichiometry $Fe_{2.6}O_4$, i.e. very close to the experimental (ideal) maghemite stoichiometry $Fe_{2.667}O_4$. As above mentioned, in the model slab, 36 Fe atoms occupied the octahedral sites, while the remaining 16 Fe were located in tetrahedral sites. In particular, the chemical stoichiometry of the system was $[Fe_{52}O_{76}(OH)_4](Si(OCH_3)_2-CH_2-CH_2-CH_2-NH_3)41(H_2O)$. The total system was electrically neutral. Octahedral site vacancies were randomly distributed in the slab, however, in line with experimental indications, vacancy close contacts were avoided. The maghemite slab (in square parenthesis in the chemical formula) is partially hydroxylated on the surface where the amino-alkoxysilane is chemisorbed. The maghemite slab was built adopting the cell parameters determined from the synchrotron X-ray diffraction experiments. The slab area was 10.234×11.817 Å^2 (in the x,y plane), and the thickness was 14 Å in the z direction.

In order to minimize interaction between images, a vacuum region of 12 Å was added along the z-direction of the slab and filled with water molecules. Hence, the simulation box consisted of $10.234\times11.817\times26.0$ Å^3. Specifically, the guess structure for the chemisorbed $-Si(OR)_2-(CH_2)_3NH_3^+$ moiety on the maghemite slab was solvated with 41 water molecules per simulation cell. Such a number of water molecules was chosen in such a way to reproduce as closely as possible the density of liquid water at ambient conditions. Periodic boundary conditions were applied in three

dimensions to the simulation cell, which contained a total of 284 atoms (52 Fe, 124 O, 101 H, 1 N, 5 C, 1 Si).

The electronic structure of the system was modeled in the framework of (Spin Polarized) Density Functional Theory (DFT). The chosen approximation was the PBE functional, combined with D2 empirical dispersion corrections (PBE-D2).[12,13] The magnetization was determined using a Hubbard Hamiltonian model, using for Fe a U parameter of 4.0 eV.[14] With this computational setup, the total magnetization (number of up-spin minus down-spin) was calculated self-consistently and resulted 86 mag/cell. Such calculated magnetization was kept fixed during the finite temperature simulation, which was performed without the Hubbard contribution to the Hamiltonian.

The electron-ion cores interaction was described with ultrasoft pseudopotentials.[15] Calculations were performed by expanding the wavefunction, at Γ point, in a plane wave basis set, and employing as planewave cutoff 30 Ry (240 Ry for electron density) and periodic boundary conditions.

The simulation approach used for studying the dynamics of this system was the Car-Parrinello First Principles Molecular Dynamics.[16] Simulations were performed in the NVT ensemble. The simulation parameters were: Target temperature of the thermostats = 298 K; integration time step = 5 au, fictitious mass for the wavefunction coefficients = 500 au. After 10 ps equilibration time, the total elapsed simulation time for the production run was 15 ps. Data were averaged and analyzed over the 15 ps trajectory. All calculations were performed with the Quantum Espresso suite of programs: in particular, the PWscf code was adopted for the self-consistent calculation of the magnetization with the Hubbard Hamiltonian, while the CP code was employed for the FPMD simulation without the Hubbard Hamiltonian but with fixed magnetization.[17]

Power spectra were calculated from the Fourier Transform of velocity autocorrelation functions (VVACF) obtained from the FPMD trajectory. Power spectra from momentum-momentum-cross-correlation functions (MMCCF)[18,19] were calculated in the same way from the same FPMD trajectory. Such cross correlations functions are typically used to monitor energy transfers among atoms from molecular dynamics simulations.[19] By invoking Linear Response Theory, one does not need to introduce, in the system Hamiltonian, the perturbation due to the AMF. This is because, the response of the system (rise in T) upon activating the perturbation (AMF) is linear. This fact has been shown in Ref. [20] where the Authors reported a linear temperature increase with the intensity of the applied field.

Supporting Figure S2

Structural data from the FPMD simulation.

Reported below are the pair distribution functions calculated for the water molecules atoms.

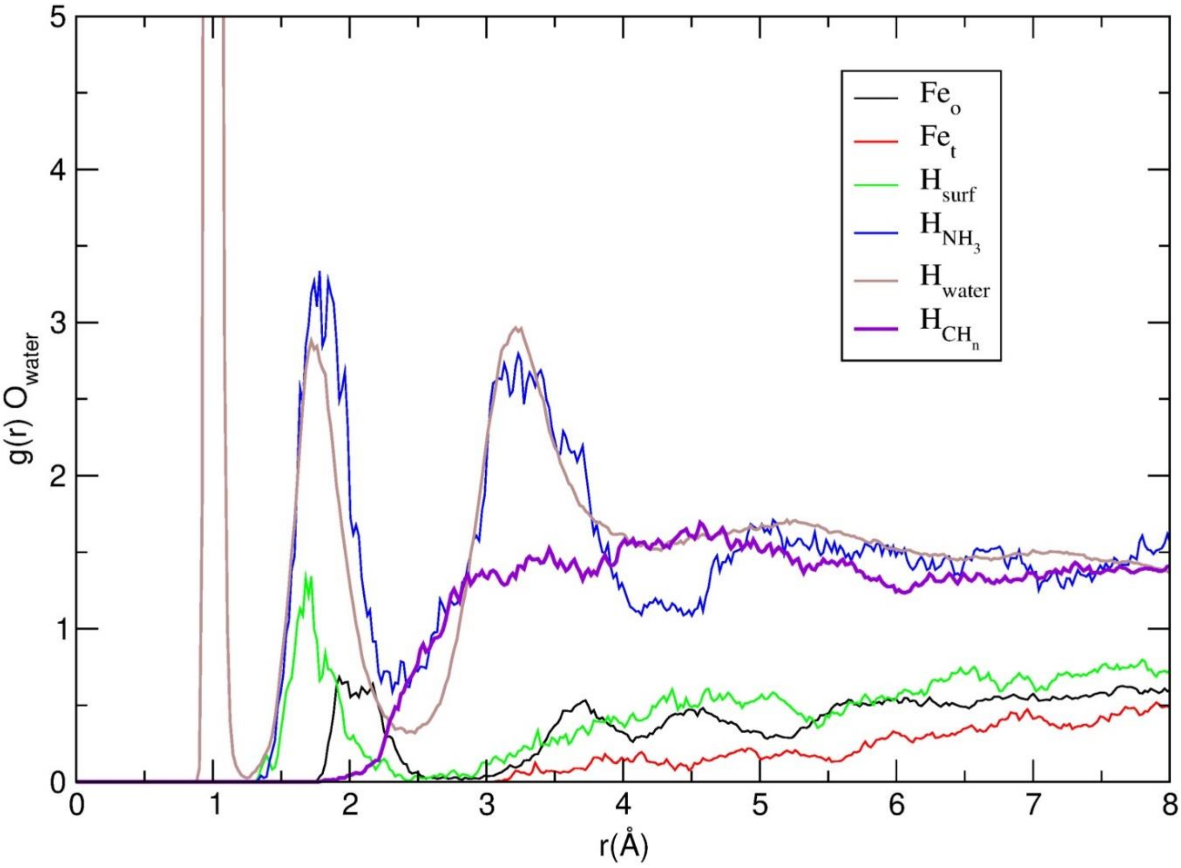

Figure S2. Calculated pair distribution functions relative to water oxygen atoms for selected contacts: octahedral Fe (Fe_o), tetrahedral Fe (Fe_t), surface protons (H_{surf}), NH_3 protons (H_{NH3}), water protons (H_{water}), and alkyl proton (H_{CHn}).

Supporting Figure S3

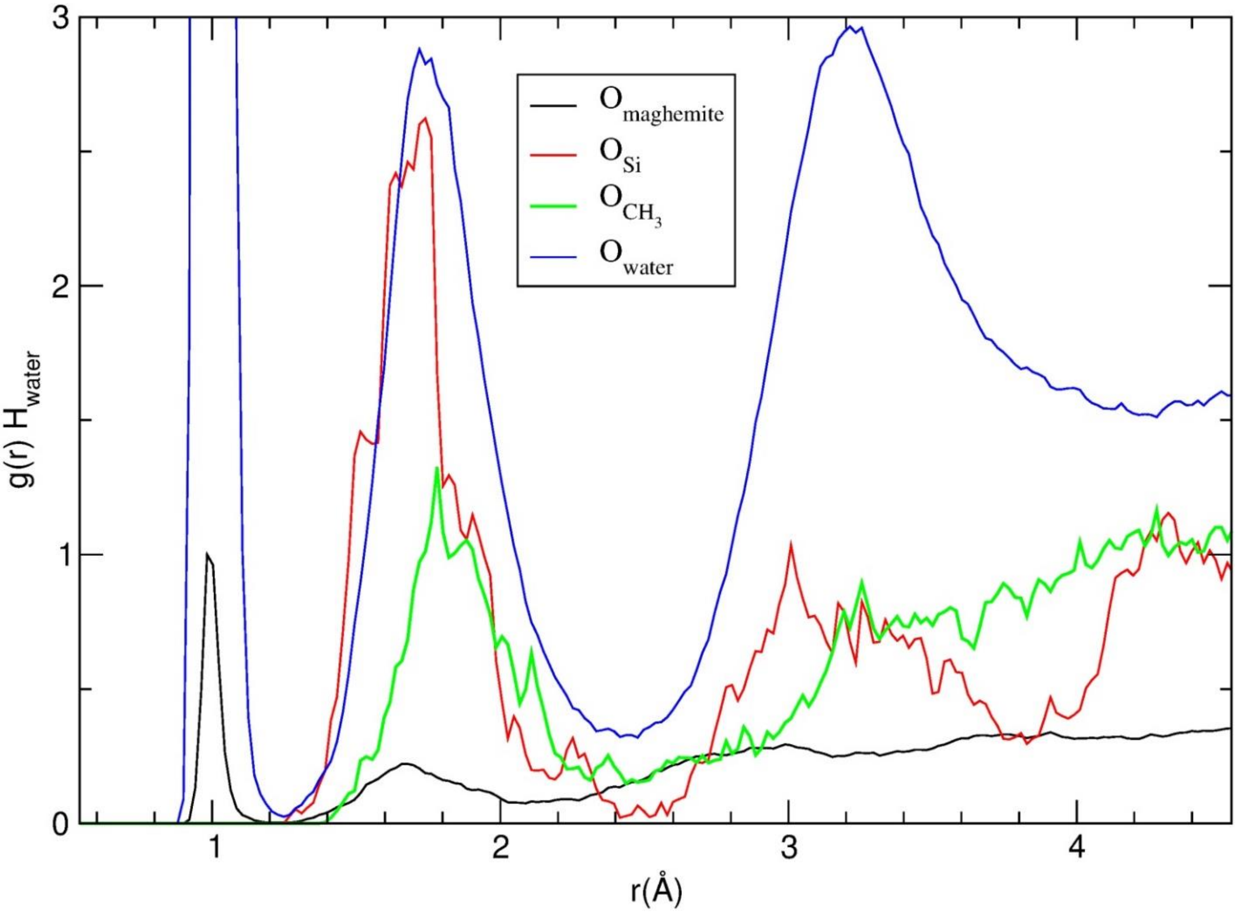

Figure S3. Calculated pair distribution functions relative to water hydrogen atoms for selected contacts: surface oxygens ($O_{maghemite}$), alkyl oxygen (O_{Si} and O_{CH3}) and water oxygens (O_{water}).

Supporting Text ST4

A comment on Figures S2 and S3

The analysis of the g(r)'s, reported in Figures S2-S3, reveals that water molecules are participating in a complex hydrogen bonding network which involves both surface and amino-alkoxy atoms, as proton acceptor (Figure S2) with respect to -NH_3 protons, surface protons and water protons. As expected, water is not involved in hydrogen bonding with the protons of the alkyl groups. O_{water} can also coordinate Fe atoms at the surface, as signaled by the broad peak centered at 2.1 Å. As proton donors (Figure S3), water molecules are engaged with surface oxygens, oxygen atoms of the alkoxysilane and obviously with water oxygen atoms. Interestingly, the peak centered at 1.0 Å in the H_{water}-$O_{maghemite}$ g(r) clearly indicates that a water molecule dissociates forming a surface O-H and an OH^- anion which in turn coordinates a surface Fe octahedral cation, whose fingerprint is included in the broad peak in the 1.9 Å-2.2 Å range of the O_{water}-Fe_o g(r) in Figure S2.

Supporting Movie

Atom Color Code: Octahedral Fe, pink; tetrahedral Fe, blue; slab O, red, H, white; water O, cyan;

Octahedral anchoring Fe, pink (large sphere), Si, yellow (large sphere); C, gray (large sphere); ethereal O, red (large sphere); N, blue (large sphere), APTES H, white (large sphere).

Supporting references

(1) Balzaretti, R.; Meder, F.; Monopoli, M. P.; Boselli, L.; Armenia, I.; Pollegioni, L.; Bernardini, G.; Gornati, R. Synthesis, Characterization and Programmable Toxicity of Iron Oxide Nanoparticles Conjugated with D-Amino Acid Oxidase. *RSC Adv* **2017**, *7* (3), 1439–1442. https://doi.org/10.1039/c6ra25349k.

(2) Mornet, S.; Grasset, F.; Portier, J.; Duguet, E. Maghemite@silica Nanoparticles for Biological Applications. *eCells and Materials Journal* **2002**, *3* (suppl. 2), 110–113.

(3) Debye, P. Zerstreuung von Röntgenstrahlen. *Ann Phys* **1915**, *351* (6), 809–823. https://doi.org/10.1002/andp.19153510606.

(4) Cervellino, A.; Giannini, C.; Guagliardi, A. DEBUSSY: A Debye User System for Nanocrystalline Materials. *Journal of Applied Crystallography* **2010**, *43* (6), 1543–1547. https://doi.org/10.1107/S0021889810041889.

(5) Frison, R.; Cernuto, G.; Cervellino, A.; Zaharko, O.; Colonna, G. M.; Guagliardi, A.; Masciocchi, N. Magnetite-Maghemite Nanoparticles in the 5-15 Nm Range: Correlating the Core-Shell Composition and the Surface Structure to the Magnetic Properties. A Total Scattering Study. *Chemistry of Materials* **2013**, *25* (23), 4820–4827. https://doi.org/10.1021/cm403360f.

(6) Cervellino, A.; Giannini, C.; Guagliardi, A. On the Efficient Evaluation of Fourier Patterns for Nanoparticles and Clusters. *J Comput Chem* **2006**, *27* (9), 995–1008. https://doi.org/10.1002/jcc.20407.

(7) Granqvist, C. G.; Buhrman, R. A. Size Distributions for Supported Metal Catalysts. Coalescence Growth versus Ostwald Ripening. *Journal of Catalysis* **1976**, *42* (3), 477–479. https://doi.org/10.1016/0021-9517(76)90125-1.

(8) Söderlund, J.; Kiss, L. B.; Niklasson, G. A.; Granqvist, C. G. Lognormal Size Distributions in Particle Growth Processes without Coagulation. *Physical Review Letters* **1998**, *80* (11), 2386–2388. https://doi.org/10.1103/PhysRevLett.80.2386.

(9) Kiss, L. B.; Söderlund, J.; Niklasson, G. A.; Granqvist, C. G. New Approach to the Origin of Lognormal Size Distributions of Nanoparticles. *Nanotechnology* **1999**, *10* (1), 25–28. https://doi.org/10.1088/0957-4484/10/1/006.

(10) Ali-Zade, R. A. Size Distribution Function of Magnetite Nanoparticles in Disperse Systems. *Inorganic Materials* **2008**, *44* (10), 1105–1109. https://doi.org/10.1134/S0020168508100154.

(11) Nelder, J. A.; Mead, R. A Simplex Method for Function Minimization. *The Computer Journal* **1965**, *7* (4), 308–313. https://doi.org/10.1093/comjnl/7.4.308.

(12) Perdew, J. P.; Burke, K.; Ernzerhof, M. Generalized Gradient Approximation Made Simple. *Phys Rev Lett* **1996**, *77* (18), 3865–3868. https://doi.org/10.1103/PhysRevLett.77.3865.

(13) Grimme, S. Semiempirical GGA-Type Density Functional Constructed with a Long-Range Dispersion Correction. *Journal of Computational Chemistry* **2006**, *27* (15), 1787–1799. https://doi.org/10.1002/jcc.20495.

(14) Cococcioni, M.; De Gironcoli, S. Linear Response Approach to the Calculation of the Effective Interaction Parameters in the LDA+U Method. *Phys Rev B Condens Matter Mater Phys* **2005**, *71* (3), 035105. https://doi.org/10.1103/PhysRevB.71.035105.

(15) Vanderbilt, D. Soft Self-Consistent Pseudopotentials in a Generalized Eigenvalue Formalism. *Physical Review B* **1990**, *41* (11), 7892–7895. https://doi.org/10.1103/PhysRevB.41.7892.

(16) Car, R.; Parrinello, M. Unified Approach for Molecular Dynamics and Density-Functional Theory. *Phys Rev Lett* **1985**, *55* (22), 2471–2474. https://doi.org/10.1103/PhysRevLett.55.2471.

(17) Giannozzi, P.; Baroni, S.; Bonini, N.; Calandra, M.; Car, R.; Cavazzoni, C.; Ceresoli, D.; Chiarotti, G. L.; Cococcioni, M.; Dabo, I.; Dal Corso, A.; De Gironcoli, S.; Fabris, S.; Fratesi, G.; Gebauer, R.; Gerstmann, U.; Gougoussis, C.; Kokalj, A.; Lazzeri, M.; Martin-Samos, L.; Marzari, N.; Mauri, F.; Mazzarello, R.; Paolini, S.; Pasquarello, A.; Paulatto, L.; Sbraccia, C.; Scandolo, S.; Sclauzero, G.; Seitsonen, A. P.; Smogunov, A.; Umari, P.; Wentzcovitch, R. M. QUANTUM ESPRESSO: A Modular and Open-Source Software Project for Quantum Simulations of Materials. *Journal of Physics Condensed Matter* **2009**, *21* (39), 395502. https://doi.org/10.1088/0953-8984/21/39/395502.

(18) Balucani, U.; Lee, M. H.; Tognetti, V. Dynamical Correlations. *Physics Report* **2003**, *373* (6), 409–492. https://doi.org/10.1016/S0370-1573(02)00430-1.

(19) Verdaguer, A.; Padró, J. A.; Trullàs, J. Molecular Dynamics Study of the Velocity Cross-Correlations in Liquids. *Journal of Chemical Physics* **1998**, *109* (1), 228–234. https://doi.org/10.1063/1.476555.

(20) Riedinger, A.; Guardia, P.; Curcio, A.; Garcia, M. A.; Cingolani, R.; Manna, L.; Pellegrino, T. Subnanometer Local Temperature Probing and Remotely Controlled Drug Release Based on Azo-Functionalized Iron Oxide Nanoparticles. *Nano Lett* **2013**, *13* (6), 2399–2406. https://doi.org/10.1021/nl400188q.
